# The Identification of RPL4 as a Hub Gene Associated with Goat Litter Size via Weighted Gene Co-Expression Network Analysis

**DOI:** 10.3390/ani14101470

**Published:** 2024-05-15

**Authors:** Zhifei Zhang, Xueying Tang, Dagang Li, Xiong Tong, Li Min, Weidong Chen, Xianghong Ju, Bin Xu

**Affiliations:** 1 Key Laboratory of Animal Nutrition and Feed Science in South Chian, Ministry of Agriculture and Rural Affairs, Guangdong Provincial Key Laboratory of Animal Breeding and Nutrition, Institute of Animal Science, Guangdong Academy of Agricultural Sciences, Guangzhou 510642, China; zhangzhifei@gdaas.cn (Z.Z.); tangxueying0502@126.com (X.T.); lidagang@gdaas.cn (D.L.); tongxiong@gdaas.cn (X.T.); minli@gdaas.cn (L.M.); chenweidong@gdaas.cn (W.C.); 2 Heyuan Branch, Guangdong Laboratory for Lingnan Modern Agriculture, Heyuan 517500, China; 3 College of Coastal Agricultural Sciences, Guangdong Ocean University, Zhanjiang 524088, China

**Keywords:** goat, reproductive performance, WGCNA, oocytes, hub gene

## Abstract

**Simple Summary:**

The reproduction of goats is a highly complex and dynamic process of life regulation, which is synergistically regulated by various aspects such as central nervous system regulation, reproductive system development, oocyte maturation, and fertilized egg development. In goats, there is still an urgent need to explore marker genes related to reproductive performance. This study identified the RPL4 gene as a key marker gene related to reproductive capacity in goat oocytes through various bioinformatics analysis methods including WGCNA, PPI network analysis, and differential gene expression analysis. The results reveal that the RPL4 gene in oocytes has the potential to serve as a biological marker for determining the reproductive rate of goats, deepening our understanding of the regulatory mechanisms of goat reproductive performance.

**Abstract:**

Reproduction in goats is a highly complex and dynamic process of life regulation, involving coordinated regulation from various aspects such as central nervous system regulation, reproductive system development, oocyte maturation, and fertilized egg development. In recent years, researchers have identified numerous genes associated with goat reproductive performance through high-throughput sequencing, single-cell sequencing, gene knockout, and other techniques. However, there is still an urgent need to explore marker genes related to goat reproductive performance. In this study, a single-cell RNA sequencing dataset of oocytes (GSE136005) was obtained from the Gene Expression Omnibus (GEO) database. Weighted Gene Co-expression Network Analysis (WGCNA) was utilized to identify modules highly correlated with goat litter size. Through gene function enrichment analysis, it was found that genes within the modules were mainly enriched in adhesive junctions, cell cycle, and other signaling pathways. Additionally, the top 30 hub genes with the highest connectivity in WGCNA were identified. Subsequently, using Protein–Protein Interaction (PPI) network analysis, the top 30 genes with the highest connectivity within the modules were identified. The intersection of hub genes, key genes in the PPI network, and differentially expressed genes (DEGs) led to the identification of the RPL4 gene as a key marker gene associated with reproductive capacity in goat oocytes. Overall, our study reveals that the RPL4 gene in oocytes holds promise as a biological marker for assessing goat litter size, deepening our understanding of the regulatory mechanisms underlying goat reproductive performance.

## 1. Introduction

For a long time, mutton, goat milk, and wool products from the goat breeding industry have provided a large number of high-protein diets and high-quality natural materials for human activities. However, with the expansion of populations and the continuous improvement of consumer demand, traditional breeding methods and technologies cannot meet the market demand gradually, and the health and sustainability of goat industry development are seriously challenged. At present, trying to improve the reproductive performance of goats is considered as the main way to increase their breeding efficiency [1]. There are many factors that influence the reproductive performance of goats, including breed, age, and environment [2,3]. The evaluation of goat reproductive performance can be based on multiple indicators such as mating rate, conception rate, lambing rate, lamb survival rate, and reproductive survival rate [4]. Consequently, the level of goat reproductive performance can comprehensively reflect the quality of breeds, feeding conditions, and management practices within the farming operation [5]. It is of great significance to selectively breed individuals with high reproductive performance and timely cull those that do not meet the requirements in order to enhance farming efficiency.

Accordingly, there is currently no simple and efficient way to determine the reproductive performance of an individual goat. Traditional methods of assessment are based on observing the basic conditions of a goat after their first or second lambing [6]. In the field of quantitative genetics, Genome-Wide Association Studies (GWAS) are commonly used to identify DNA molecular markers associated with traits, thereby determining trait–marker linkage sites [7]. In humans, these markers have been effectively used in predicting human diseases [8,9]. Similarly, in goat breeding research, researchers have laid the foundation for selecting traits related to meat quality, wool quality, milk production, disease resistance, reproductive traits, and other traits using GWAS and gene chip technologies [10]. However, the exploration of key regulatory genes and signaling pathways related to reproductive performance is still incomplete, indirectly reducing the accuracy of genomic selection. Therefore, it is necessary to apply new technologies from new perspectives to improve the situation. Additionally, the exploration and identification of key genes that can be used to predict goat reproductive performance are equally important.

In this study, a series of bioinformatics analysis methods were employed to identify important genes that can serve as biological markers for goat reproductive performance, which will contribute to genetic advancements in breeding high reproductive capacity goats. Firstly, a dataset on goat oocytes was downloaded from the GEO database (GSE136005) representing high or low reproductive capacity. Secondly, the differentially expressed genes between the high and low reproductive capacity groups were analyzed and the potential biological functions and signaling pathways of these differentially expressed genes were explored. Thirdly, a gene co-expression network was constructed, and core modules related to reproductive traits and their hub genes were identified. Subsequently, Protein–Protein Interaction (PPI) network analysis was conducted using genes from the core modules and filtered out highly ranked interacting genes. Finally, by analyzing the intersection of hub genes, differentially expressed genes, and interacting genes, marker genes highly correlated with reproductive performance in goat oocytes were identified. Overall, this study utilized methods such as WGCNA and PPI analysis to identify potential marker genes associated with goat reproductive performance, which may provide a new perspective for understanding the mechanisms underlying differences in goat reproductive capacity.

## 2. Materials and Methods

### 2.1. Data Collection and Processing

The dataset related to goat oocyte single-cell RNA sequencing for reproductive traits (GSE136005) was downloaded from the GEO database (https://www.ncbi.nlm.nih.gov/geo/, accessed on 10 December 2023). This dataset includes single-cell RNA-seq data from goat oocytes, with a total of 15 samples each from high reproductive performance (litter size > 3) and low reproductive performance (litter size < 2) goats [11]. All of the experimental sheep were healthy 2–3-year-old Ji’Ning grey goats, and the breeding place was in Heze City, Shandong Province, China (35°15′4.0″ N, 115°24′38.6″ E). According to the description, the original data were compared using CellRanger (Version 6.1.2) and calculated via “UMI” to acquire the basic sample data quality information. The Vakid barcodes index of the data is over 97%, the Valid UMI index is 100%, the sequencing saturation index is over 70%, and the comparison rate with the reference genome is over 93.4%, which indicates that the data are highly reliable and can be used for subsequent analysis [11].

Subsequently, the data in the dataset were log2 transformed, and the raw count expression data were normalized using the “normalizeBetweenArrays” function from the R (Version 4.3.3) package “limma”. Specifically, this method first arranges the data values of each sample from small to large and then replaces the arranged data values with corresponding quantiles, so that the data distribution of each sample is the same and a better comparison and comprehensive analysis can be made [12].

### 2.2. Identification of DEGs

The sample data’s gene expression profiles were analyzed using the R package “edgeR” to identify differentially expressed genes (DEGs) between the high reproductive performance group and the low reproductive performance group. The criteria for DEGs were set as |log2Fold Change (log2FC)| > 1 and *p* < 0.05. The volcano plot of the DEGs was created using the R package “ggplot2”. Functional enrichment analysis and pathway analysis of all genes were conducted using the R package “clusterProfiler”, and the results were visualized using the R package “ggplot2”.

### 2.3. Construction of the Gene Co-Expression Network

The R package “WGCNA” was used to construct a gene co-expression network and identify modules related to goat reproductive traits [13]. In order to exclude extremely low or meaningless gene expression data, the standard deviation of all gene expression in the dataset was calculated, and the genes were sorted according to the standard deviation, and then, the top 5000 genes were extracted for further analysis. Second, the “hclust” function was used to cluster the samples, and outlier samples were removed (2 samples were excluded). Third, the correlation between each pair of genes was calculated to obtain a gene similarity matrix, which was then transformed into an adjacency matrix. Fourth, using the “pickSoftThreshold” function, a soft threshold was chosen based on the criterion of an unscaled network fitting index R^2^ > 0.9. Fifth, the “adjacency” function was used to convert the adjacency matrix into a topological overlap matrix (TOM). Sixth, module–trait relationship analysis was performed, revealing that the target module was the most important module related to reproductive traits.

Subsequently, the module significance (GS) and module membership (MM) of genes within the module were calculated and sorted in descending order. The top 30 genes with the highest connectivity within the module were selected as hub genes for further analysis.

### 2.4. Construction of Protein–Protein Interaction (PPI) Network

The String website (https://cn.string-db.org, accessed on 20 December 2023)) was used to construct a PPI network. Using Cytoscape software (Version 3.10.2) [14], the top 30 genes ranked by node degree in the PPI network were selected as core genes. The hub genes obtained from the target module were then intersected with the core genes, resulting in the identification of target genes within the module that corresponded to the reproductive trait.

## 3. Results

### 3.1. Identification of Litter Size-Related DEGs in Goat Oocytes

**Figure 1:**
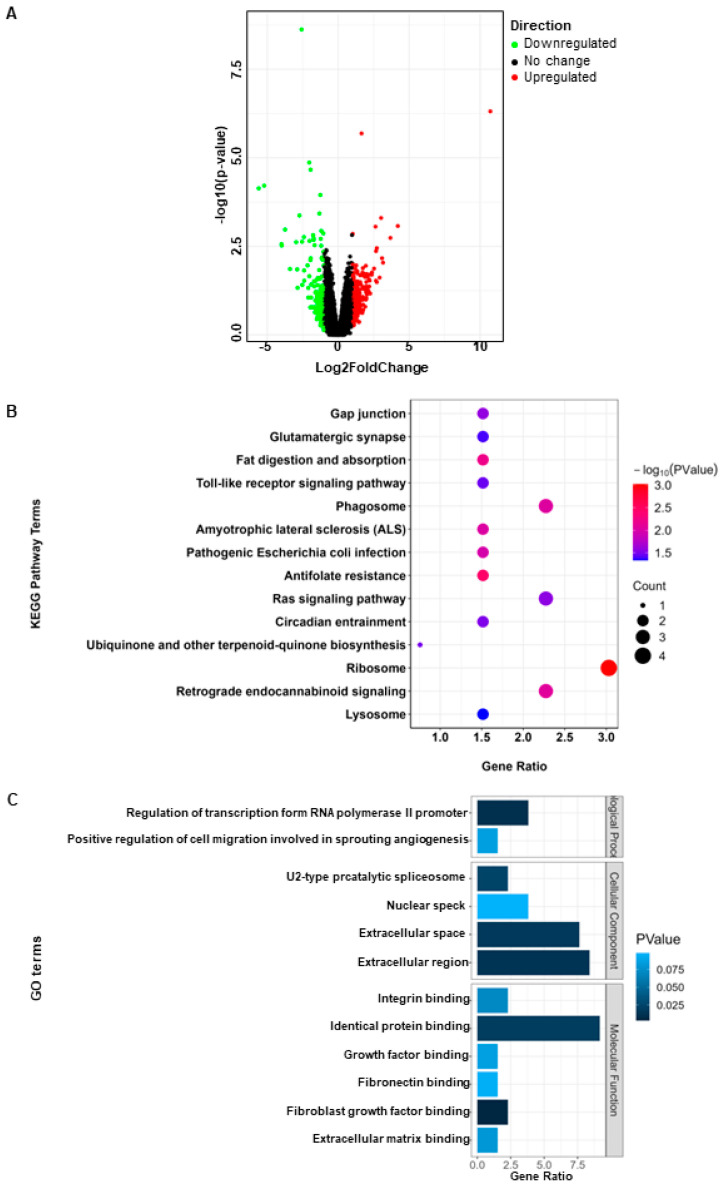
Identification and functional enrichment analysis of differentially expressed genes. (**A**) Volcano plot of DEGs; (**B**) KEGG enrichment analysis of DEGs; (**C**) GO enrichment analysis of DEGs.

After analysis, a total of 132 differentially expressed genes were identified (|log2FC| > 1, *p* < 0.05), among which 74 genes were upregulated and 58 genes were downregulated (Figure 1A). The top 10 upregulated and downregulated genes among the differentially expressed genes are shown in Table 1. Functional analysis of the differentially expressed genes revealed enrichment in several key signaling pathways including ribosome, phagosome, the Ras signaling pathway, and retrograde endocannabinoid signaling (Figure 1B). The main functions enriched include identical protein binding, extracellular region, and regulation of transcription from RNA polymerase 2 promoter (Figure 1C).

### 3.2. Weighted Gene Co-Expression Network Construction

**Figure 2:**
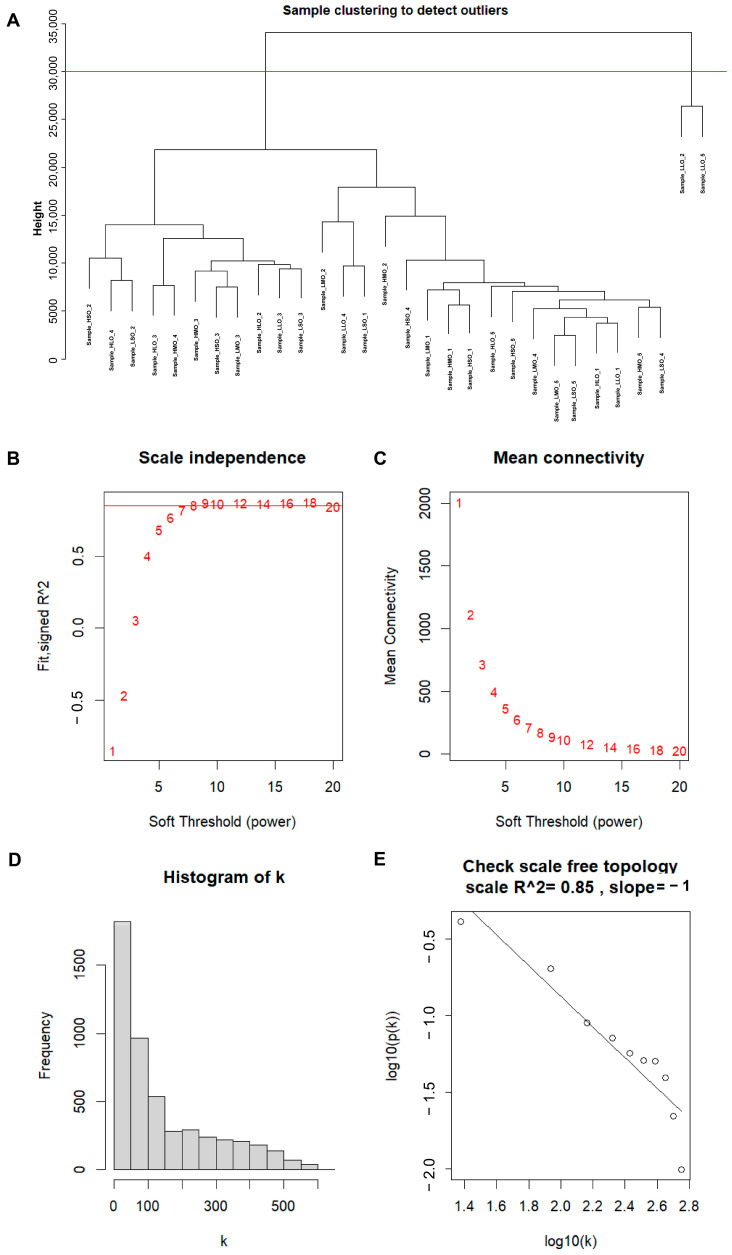
Determination of optimal soft threshold power for scale-free topology. (**A**) Sample clustering tree, with red lines indicating the dividing lines of excluded samples; (**B**) the scale-free topology fit index (R^2^) for soft threshold power from 1 to 20; (**C**) the mean connectivity (k) for soft threshold power from 1 to 20; (**D**) the mean connectivity (k) for soft threshold power from 1 to 20; (**E**) the scale-free topology R^2^ between log10 (k) and log10 (p(k)) when β = 8.

**Figure 3:**
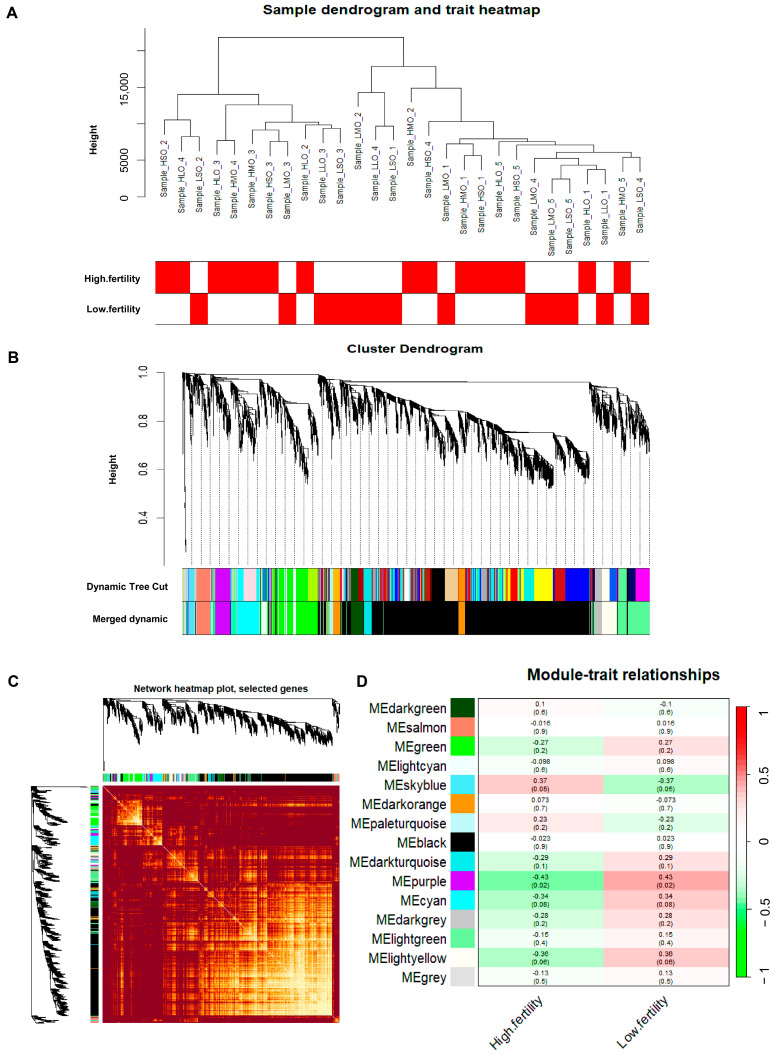
Identification of gene co-expression network modules related to goat litter size. (**A**) Sample dendrogram and trait heatmap; (**B**) clustering of module eigengenes; (**C**) network heatmap plot; (**D**) module–trait relationships.

Using Weighted Gene Co-expression Network Analysis (WGCNA) to construct a gene co-expression network, modules related to goat reproductive traits were identified. First, sample clustering was performed (Figure 2A), and two outlier samples were removed. Second, a similarity matrix was calculated, and a soft threshold of nine was selected as the optimal value, followed by transformation into an adjacency matrix (Figure 2B,C). A reverse proportional relationship between p(k) and k was observed (Figure 2D), with an unscaled topological R^2^ of 0.85 for log10 (p(k)), indicating that a soft threshold of 9 was suitable for constructing an unscaled topological network (Figure 2E). Based on topological overlap, similar genes were hierarchically clustered and dynamically cut, resulting in the identification of different modules (Figure 3A–C). Third, correlation analysis was performed between module eigengenes and phenotype information. As shown in Figure 3D, a module named MEpurple was identified, consisting of 162 genes, which showed a negative correlation with high reproductive traits (r = −0.43; *p* = 0.02).

### 3.3. Screening and Functional Enrichment Analysis of Hub Gene in MEpurple Module

**Figure 4:**
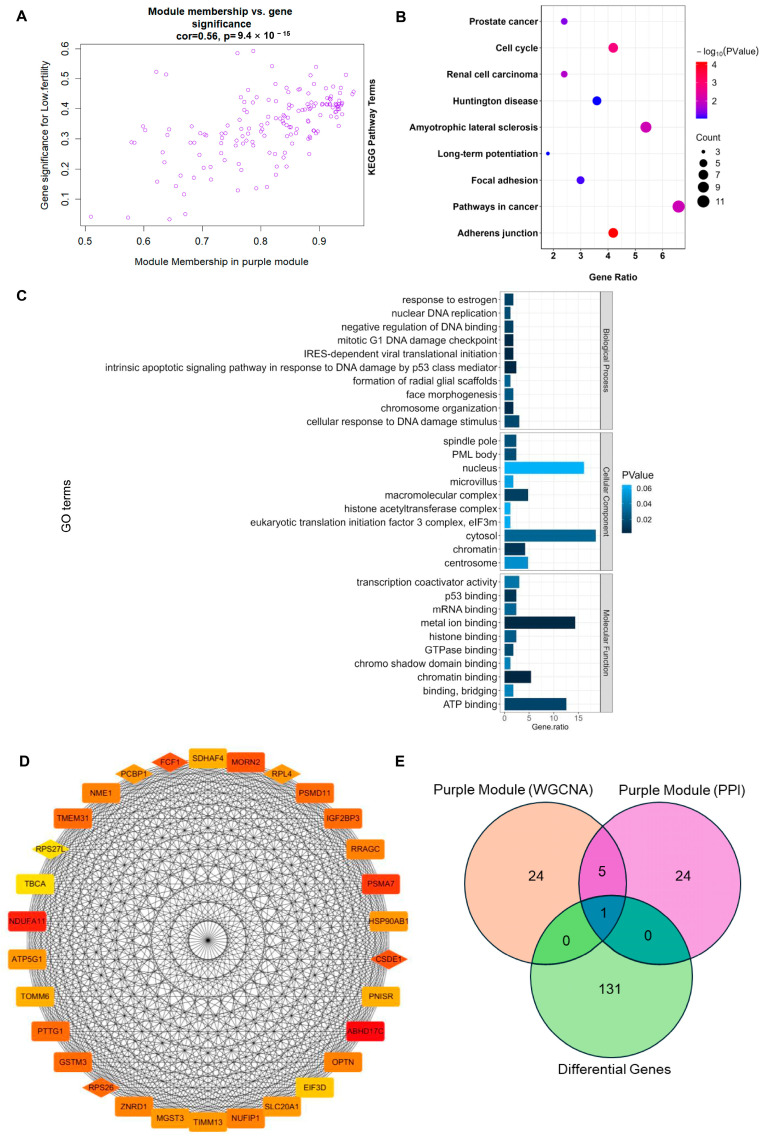
Identification of marker genes related to goat litter size. (**A**) Scatterplot of module membership (MM) in the MEpurple module versus GS for litter size; (**B**) KEGG enrichment analysis of genes in the MEpurple module; (**C**) GO enrichment analysis of genes in the MEpurple module; (**D**) for the hub gene in the purple module and the core gene in the PPI analysis, the darker the color, the higher the correlation between the genes, and the diamond indicates the shared gene; (**E**) Venn diagram analysis between hub genes of the purple module, core genes of the PPI analysis, and DEGs.

The analysis within the module showed a close relationship between module membership (MM) and gene significance (GS) in the MEpurple module (correlation = 0.56, *p* < 0.001; Figure 4A). Functional enrichment analysis of genes in the ME purple module revealed enrichment in key signaling pathways such as adherens junction, cell cycle, and pathways in cancer (Figure 4B), as well as enrichment in main functions including metal ion binding, ATP binding, cytosol, and nucleus (Figure 4C).

### 3.4. Identification of RPL4 as a Hub Gene Associated with Goat Litter Size

The top 30 genes with the highest correlation between GS and MM in the ME purple module were selected as hub genes (Figure 4D). Among these hub genes in the MEpurple module and the top 30 core genes related to protein interactions identified through Protein–Protein Interaction (PPI) network analysis, six target genes related to reproductive traits were obtained: RPS26, RPS27L, CSDE1, PCBP1, FCF1, and RPL4 (Figure 4D). Comparing the differentially expressed genes, the hub genes in the purple module, and the core genes from the PPI analysis, it was found that the RPL4 gene appeared in all three sets (Figure 4E). Therefore, it was identified as a marker gene related to goat reproductive performance in this study.

## 4. Discussion

The quality of oocytes is a key determinant of mammalian reproductive performance, directly impacting the quantity of high-quality embryos and pregnancy rates [15,16]. Although it has been reported that oocyte function is jointly regulated by genetic factors and nutritional metabolism [17], the regulatory mechanisms remain unclear. Reproductive performance in goats is one of the most important traits in the goat breeding process, making the enhancement of goat population reproductive performance a focal point of recent attention. Identifying marker genes related to reproductive performance in oocytes and elucidating their potential regulatory mechanisms may help quickly identify individuals with high reproductive performance, thereby increasing the efficiency of enhancing population reproductive performance. In this study, the sequencing data of oocytes from goats with high and low reproductive performance were systematically analyzed. Through WGCNA and PPI network analysis, potential marker genes were identified that serve as determinants of goat reproductive performance and elucidated the reproductive trait-related regulatory pathways in oocytes.

Reproductive traits are complex quantitative traits encompassing important indicators such as litter size and ovulation rate, which are regulated by major effect genes and multiple minor effect genes, often referred to as reproductive genes [18,19]. Through traditional molecular marker techniques such as restriction fragment length polymorphism (RFLP), microsatellites, and single nucleotide polymorphisms (SNPs), some major effect genes such as *progesterone receptor* (*PGR*), *estrogen receptor* (*ESR*), *follicle-stimulating hormone* (*FSHR*), *prolactin receptor* (*PRLR*), etc., have been identified [20,21,22]. It is not difficult to find that these genes are more or less related to the regulation function of the neuroendocrine–reproductive system and have significant impacts on reproductive performance. Among the differential genes analyzed in this study, *Limbic System-Associated Membrane Protein* (*LSAMP*), and *Neuron Navigator 3* (*NAV3*) are associated with neurodevelopment [23,24], *Phospholipase A2 group* X (*PLA2G10*), *Discs large MAGUK scaffold protein 2* (*DLG2*), *Serpin Peptidase Inhibitor, clade A* (*alpha-1 antiproteinase*, *antitrypsin*)*,* and *member 14* (*SERPINA14*) can regulate reproductive cell function [25,26,27], while *Keratin 23* (*KRT23*), TIMP metallopeptidase inhibitor 1 (*TIMP1*), and TNF alpha-induced protein 6 (*TNFAIP6*) have been reported to be involved in the regulation of immune function and inflammatory responses [28,29,30]. 

Kimura et al. [31] conducted a comprehensive investigation into the precise expression patterns of *LSAMP* during early embryogenesis. Their findings revealed that *LSAMP* expression was predominantly localized to specific regions of the brain and neural crest. Particularly within the developing neural tube, *LSAMP* expression was observed in the anterior midbrain. These results underscore the critical roles of *LSAMP* during embryogenesis, particularly in the processes of brain formation and differentiation. Another noteworthy gene identified in this study is *SERPINA14*, which was initially characterized in the 1980s as ovine uterine serpin (OvUS), a major secretory protein expressed in the endometrium during pregnancy in ruminants [32,33]. In the bovine endometrium, *SERPINA14* mRNA levels were notably higher during estrus, coinciding with elevated estrogen levels, compared to the P4-dominated diestrus phase [34]. Moreover, a comparison between cyclic and pregnant endometrium on day 18 revealed the upregulation of *SERPINA14* during the preimplantation phase despite similar levels of P4 [35]. These studies not only underscore the significance of *SERPINA14* expression as a biomarker for characterizing estrus and pregnancy in cattle but also highlight its potential as a therapeutic target for enhancing reproductive performance in animals.

The functional enrichment analysis results show that the main function of differential genes is related to the “Regulation of transcription from RNA polymerase 2 promoter”, which has been reported to be involved in the activation of the early embryonic genome, as described previously [36]. It is believed that it is not sufficient to only look for marker genes related to goat reproductive performance from the perspective of differential genes, because the important role of some genes may not only be directly changed through transcriptional level changes but may also be jointly regulated by changes in multiple genes. The differential expression analysis method still has limitations in finding target trait-related polygenic minor-effect genes. Therefore, WGCNA was used to identify gene modules related to goat reproductive traits (ME purple module) and selected reproductive-related hub genes from the purple module. 

Animal litter size is a complex quantitative trait involving various processes including hormone secretion, follicular development, ovulation, fertilization, embryo implantation, placental and fetal development, etc. [37]. Classical genetics postulates that quantitative traits are governed by numerous minor-effect polygenes, often regulated by major-effect genes and numerous minor-effect genes. Currently, several major-effect genes related to sheep reproductive traits have been identified, including *Bone morphogenetic protein receptor 1B* (*BMPR1B*), *Bone morphogenetic protein 15* (*BMP15*), and *Growth differentiation factor 9* (*GDF9*), and some minor-effect genes such as *Prolactin receptor* (*PRLR*) and *Follicle stimulating hormone receptor* (*FSHR*), whose expression in animals often coincides with reproductive patterns, and their genetic variations significantly influence reproductive performance [38,39,40]. In this study, the method of WGCNA precisely delineates and identifies modules functionally and expressionally associated with goat litter size traits. Furthermore, it is imperative to delve deeper into how the identified modular genes impact the pregnancy rates and overall reproductive performance of animals. This time, through functional enrichment analysis of genes within the module, more specific key signaling pathways related to goat reproductive performance have been elucidated, such as adherens junction, cell cycle, metal ion binding, ATP binding, etc., which have been reported in other animal species to be crucially associated with oocyte development and function [41].

To further elucidate the gene interaction network within the MEpurple module, in the study, we performed PPI analysis on the 164 genes in this module and selected the top 30 genes with the highest interaction ranks. Subsequently, by taking the intersection of hub genes, core genes in protein interactions, and DEGs, the *Ribosomal protein L4* (*RPL4*) was ultimately identified as a key marker gene related to goat reproductive performance. Previous studies have shown that the *RPL4* gene, as a protein-coding gene, plays an important role in peptide chain elongation, rRNA processing, and synthesis and is involved in functions such as RNA binding and ribosome activity. Research by Mo et al. shows that RPL4 located in the mitochondria plays a unique regulatory role in mitochondrial activity and the Notch signaling pathway [42]. Deletion of *RPL4* leads to decreased mitochondrial activity and interacts with the Wap–mnb complex, affecting the binding of the transcription factor Su(H) with chromatin, thus exerting significant effects on the growth and development of Drosophila and zebrafish [42]. Research by He et al. indicates that the loss of *RPL4* can induce ribosomal stress, and its stable expression helps regulate the MDM2-p53 signaling loop, which is crucial for normal cell growth and proliferation [43]. It is evident that the oocytes used in this study not only need to maintain the stability of genetic material but also need to meet the biological needs of subsequent embryo growth and development. Therefore, the maintenance of mitochondrial activity and the regulation of development-related gene function are crucial for their reproductive performance. Thus, this is reason to speculate that the RPL4 gene, selected through various bioinformatics analysis methods in this study, has great potential as a marker gene for determining the high or low reproductive performance of goats. 

However, there are still some limitations in the current study. Firstly, due to the limited number of databases related to goat reproductive traits, the sample size selected in this study was less than 15 in each group, and the data were from a single breed of goats, which may reduce the accuracy of identifying marker genes. Despite this, this study approached the analysis from three aspects: DEG analysis, WGCNA, and PPI network analysis, with the aim of increasing the reliability of the results obtained. The focus was not on obtaining a large number of marker genes but on the quality of the marker genes obtained through step-by-step screening. Considering existing reports, the ultimately selected marker gene *RPL4* is considered the most important. Secondly, the dataset used in this study mainly reflects the reproductive trait of litter size in goats. Although litter size can correspond well with oocyte function, in future studies, we will also focus on other reproductive traits such as intrapartum interval, mating rate, and viability rate. Finally, it is noteworthy to mention that this study relies solely on bioinformatics methodologies, lacking supplementary independent animal or cell verification tests. Consequently, there exists a limitation in identifying potentially significant genes as marker genes, thereby impacting the confidence level of the current findings to some extent. Likewise, independent functional validation of the key gene *RPL4* is absent in this study. The authors’ future endeavors will prioritize a comprehensive assessment of *RPL4’*s role and mechanism as a marker gene for goat litter traits, employing alternative technical methodologies such as gene overexpression or knockout experiments. This strategic approach aims to strengthen the validity and robustness of the study’s conclusions.

In summary, based on the gene expression profiles of the single-cell sequencing of oocytes from goats with different reproductive capacities, in this study, we conducted a comprehensive bioinformatics analysis of marker genes related to goat reproductive traits. Through the combined analysis of differential gene expression, WGCNA, and PPI networks, the potential marker gene *RPL4* for judging the high or low reproductive capacity of goats was identified. Additionally, signaling pathways such as adherens junction, cell cycle, metal ion binding, and ATP binding may be key mechanisms through which goat oocytes influence goat reproductive traits.

## Figures and Tables

**Table 1:** To 10 upregulated/downregulated DEGs.

Terms	High Litter Size Group vs. Low Litter Size Group
Upregulated genes (top 10)	*LSAMP*; *TMEM80*; *DEFB116*; *SMCO4*; *LOC102187672*; *DLG2*; *PLA2G10*; *LOC102179185*; *GNG11*; *LOC102170446*
Downregulated genes (top 10)	*NAV3*; *ENSCHIG00000010736*; *KRT23*; *SLC10A6*; *TIMP1*; *THBS1*; *SERPINA14*; *TNFAIP6*; *RPS29*; *TMSB4X*

## Data Availability

The data from this study were deposited in the GEO database (https://www.ncbi.nlm.nih.gov/geo/, accessed on 10 December 2023) under the accession number PRJNA560927.
